# Measuring and Improving What Really Matters: A Population Health Solution for Aging Well

**DOI:** 10.1093/geroni/igaf122.529

**Published:** 2025-12-31

**Authors:** James Firman

**Affiliations:** BetterAge, Arlington, Virginia, United States

## Abstract

Traditional health and social service metrics often fail to capture the full picture of what enables older adults to age well. BetterAge is pioneering a new approach—one that measures holistic health and wellbeing across 13 dimensions, identifies 17 critical barriers, and provides hyper-personalized recommendations to drive meaningful action. The session will explore how BetterAge integrates emerging technologies with trusted community networks to assess, engage, and support older adults at scale. Supported by a $2.5M SBIR Fast Track grant from the National Institute on Aging, BetterAge leverages AI-driven assessments, behavioral insights, and strategic partnerships, including USAging and state-level initiatives, to generate real-world impact. Recent pilots with community organizations and state agencies through the Age Well Innovation Network (AWIN) are beginning to demonstrate its potential to identify disparities, get people to change behaviors known to improve outcomes, and reduce social isolation. Participants will learn how this population health solution is being implemented nationwide, how it aligns with evolving policy and funding landscapes, and how organizations can leverage it to support older adults more effectively. The session will include real-world case studies, data-driven insights from 4,000+ consumer records, and a discussion on the future of measuring what truly matters for aging well.

